# Safety and Efficacy of Cell Transplantation on Improving Motor Symptoms in Patients With Parkinson’s Disease: A Meta-Analysis

**DOI:** 10.3389/fnhum.2022.849069

**Published:** 2022-05-06

**Authors:** Jiaming Wang, Yu Tian, Xin Shi, Zhaohai Feng, Lei Jiang, Yujun Hao

**Affiliations:** ^1^Department of Neurosurgery, First Affiliated Hospital of Xinjiang Medical University, Ürümqi, China; ^2^Department of Neurosurgery, Shenzhen University General Hospital and Shenzhen University Clinical Medical Academy Centre, Shenzhen University, Shenzhen, China

**Keywords:** Parkinson’s disease, stem cells, cell transplantation, motor, meta-analysis

## Abstract

**Background:**

The past four decades have seen the growing use of tissue or cell transplants in Parkinson’s disease (PD) treatment. Parkinson’s cell therapy is a promising new treatment; however, efficacy of cell transplantation for Parkinson’s disease are entirely unclear.

**Objective:**

To conduct a meta-analysis and a systematic review of the efficacy of cell therapy in patients with PD.

**Methods:**

A systematic literature review and meta-analysis of 10 studies were performed to assess the efficacy of cell therapy in Parkinson’s patients. To achieve this, we compared the change in Unified Parkinson’s Disease Rating Scale (UPDRS) II and III scale scores to baseline and assessed the incidence of transplant-related adverse events. The MINORS score and the I^2^ index were applied to evaluate the quality of studies between-study heterogeneity, respectively.

**Results:**

The literature search yielded 10 articles (*n* = 120). The improvement in motor function based on the UPDRSIII assessment was −14.044 (95% CI: −20.761, −7.327) (*p* < 0.001), whereas improvement in daily living ability based on the UPDRSII assessment was −5.661 (95% CI: −7.632, −3.689) (*p* < 0.001).

**Conclusion:**

The present findings demonstrate important clues on the therapeutic effect of cell therapy in alleviating motor impairment and daily living ability in PD patients.

## Introduction

Parkinson’s disease (PD), the second most common neurodegenerative disease after Alzheimer’s disease, is characterized by prodromal symptoms, motor difficulties, and psychological or cognitive problems. The median age-standardized annual incidence rate of PD is 14 per 100,000 people, 160 per 100,000 people aged 65 years or older in high-income countries. For Asians, the incidence rate of PD is 11.3 per 100,000 people (Ascherio and Schwarzschild, 2016). Of note, PD has become more widespread in view of the aging population.

As a progressive neurodegenerative disease, PD causes focal degeneration of midbrain dopamine (MesDA) neurons. This reduces the release of the midbrain dopamine neuron cytosol of PD, localized in the ventral midbrain (Vm) in the substantia nigra densa, from associated axons and spiny neurons in the striatum (Poulin et al., 2018). Aside from the main PD medications, including dopamine agonists, monoamine oxidase B inhibitors, levodopa plus carbidopa, etc., anticholinergics and amantadine are also indicated in some patients (Zeuner et al., 2019; Armstrong and Okun, 2020). Off-target effects and poor targeting effects of systemic administration limit the use of drugs. Major drawbacks include poor response to current therapy for many clinically important conditions and increased dose and dose frequency with disease progression and worse symptoms, increasing adverse events.

In addition, surgical procedures such as pallidotomy or thalamotomy, or deep brain stimulation can be used to manage PD (Martínez-Fernández et al., 2018; Krauss et al., 2021). Unfortunately, most of the existing methods of therapy are aimed at symptomatic treatment and do not address the basic causes of the pathologies. Therefore, one of the most important tasks of modern neuroscience is to study the molecular mechanisms underlying neurodegeneration and search for new targets for the treatment of neurodegenerative pathologies (Grekhnev et al., 2022). Cell replacement therapy holds great promise in managing Parkinson’s disease as this condition attacks a single cellular area of the brain. Cell replacement therapy can restore the “normal” physiological pattern of striatal DA transmission. Over the past four decades, researchers have been looking for alternative strategies to supplement DA by replacing the dopaminergic neurons lost in the disease with stem-cell-derived equivalents. Embryonic Stem Cells (ESCs), Mesenchymal Stem Cells (MSCs), Neural Stem Cells (NSCs), and Induced Pluripotent Stem Cells (iPSCs) are among the stem cell typologies that scientists are working with to induce their differentiation into the mature dopaminergic cell (Sivandzade and Cucullo, 2021). Several pieces of evidence have demonstrated the potential role of transplantation of cells into animal models of neurodegenerative diseases to improve endogenous neuronal function by mediating myelin regeneration, releasing trophic factors, and modulating inflammation (Ager et al., 2015; Burns and Quinones-Hinojosa, 2021).

In view of the above reports, cellular therapy presents more benefits compared to traditional treatment modalities as it improves the continuity of drug therapy, shortens the off-time, reduces the severity of movement disorders, and delay drug-related complications associated with oral medications (Olanow et al., 2006; Seppi et al., 2019).

## Methods

### Search Strategy and Selection Criteria

The present systemic review was performed following guidelines of the Preferred Reporting Items for Systematic Reviews and Meta-Analyses (PRISMA) 2009 and the PRISMA 2020 updated statement. The requirements of an Institutional Review Board approval or informed patient consent were waived for this review. A literature search was performed in English on PubMed (MEDLINE) and Web of Science databases with no time restrictions. The search string was built as follows: (Parkinson) AND (cell transplantation). A manual search of the reference lists supplemented the electronic database search. The reference list of all selected articles was screened independently to identify additional studies that may have been missed in the initial search.

The study question was formulated according to the Population, Intervention, Comparison, Outcome, and Study design (PICOS) strategy. Studies that met the following inclusion criteria were analyzed: PD patients receiving stem cell therapy (Population), Stem cells with different cell types and different ways of administration (Intervention), Placebo control (Comparison), UPDRS II and III score staging changes from baseline (Outcome). All study designs were eligible for inclusion. However, animal experimental studies, letters, comments, and editorials were excluded. Care was taken to avoid data duplication. In the first screening phase, all studies were evaluated for eligibility based on a review of the title and the abstract. In the second screening phase, full-text articles were reviewed for eligibility criteria.

### Data Extraction

Three investigators reviewed all articles independently. The investigators consulted amongst themselves in cases of differing opinions and if agreement could not be reached other experts were consulted. The data extracted from each study included the following: first author, publication year, study type, sample size, age, course of PD, dose, route of administration, type of administration, follow-up time, treatment-related complications, UPDRS score, UPDRS improvement rate, results, and conclusions. Cross-checking of the data was performed before analysis.

### Quality Assessment of Include Studies

One author evaluated the methodological quality of all studies. Two authors held discussions and verification to achieve consensus and establish an agreement with overall rating scores. Three investigators assessed the quality of the included studies independently with the Strengthening the Methodological Index for Non-randomized Studies (MINORS) method. Notably, items were scored 0 if not reported; 1 if the reporting was inadequate; 2 if the reporting was adequate. The global ideal score was 24 and 16 for comparative and non-comparative studies. The final assessment result was decided after the discussion. None of the included studies was a comparative in nature; therefore, the maximum score was 16.

### Statistical Analysis

Meta-analyses were performed by R software 4.1.1 (R Foundation for Statistical Computing, Vienna, Austria) with Meta package (version 4.18-2) and the R package “Meta” to establish changes in UPDRS scores before and after the intervention. Results were presented as proportions at 95% confidence intervals (CIs). Forest plots were applied to illustrate the improvement of dyskinesia in PD patients by cell therapy. I^2^ and Cochrane’s Q statistics were applied to evaluate the heterogeneity among studies. For I^2^ < 40%, a fixed-effects approach was employed. Otherwise, the between-study variance was determined by the random-effects method. The Begg test was used to test for publication bias. In case of publication bias, trim and fill analysis was performed to evaluate the number of missing studies, impute missing studies and recalculate the pooled risk estimate.

## Results

### Study Selection

**FIGURE 1:**
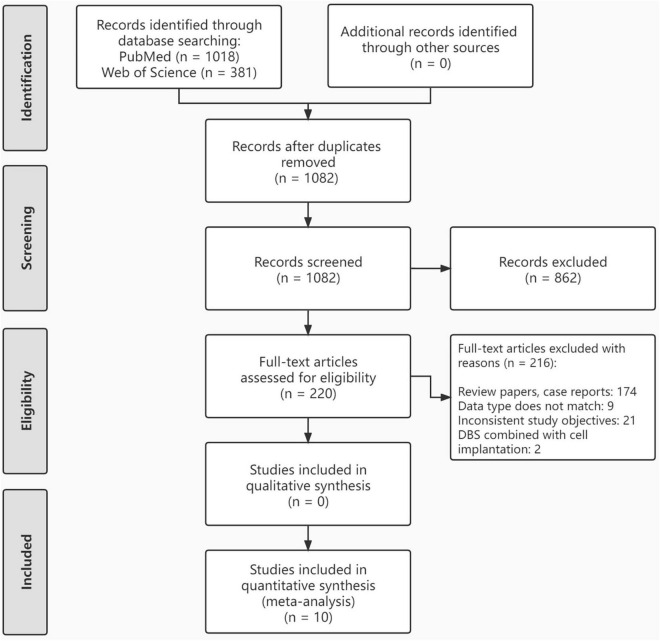
Flow chart of the study selection.

**FIGURE 2:**
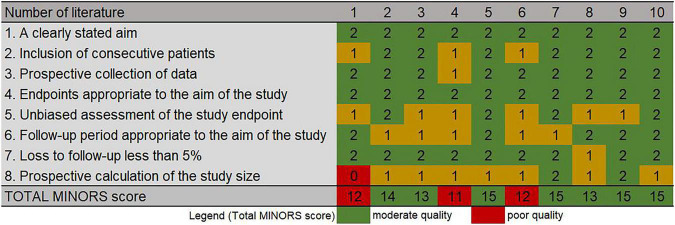
Risk of bias graph for each included study.

The literature search yielded 1,082 articles after eliminating duplicates. In the first screening phase, 862 articles were excluded, leaving 220 articles that underwent full-text review. In the second screening phase, 206 articles were excluded. As a result, 10 articles reporting on cell therapy treatment in patients with PD were included for analysis. The study selection process is illustrated in a PRISM flowchart (Figure 1). The Results of the literature quality assessment are presented in Figure 2.

### Study Characteristics

Table 1 shows the characteristics of the articles included in the final literature review. The 10 included studies were conducted between 1999 and 2019, comprising 120 patients with Parkinson’s disease, who received cell therapy. Five types of cell therapy were involved, including the human fetus, pig embryo, BM-MSCs, HRPE, and NPC. All studies adopted stereotactic transplantation. Seven studies reported significant improvement in UPDRS III, 2 studies showed poor results. In terms of safety, 7 papers reported that cell transplantation was safe and well-tolerated, whereas 3 papers reported cell transplantation-related complications, including depressive suicidal tendencies, cell transplantation-related cerebral infarction (with sequelae), and cell transplantation procedure-related death (Table 1).

**TABLE 1:** Characteristics of the articles included in the final literature review.

														
No.	Author (Published year)	Study type	Ages (yr.)	No. patients	Disease duration (yr.)	Dose	Route of administration	Type of administration	Follow-up time	AES	UPDRS (Mean ± SD)	ADL (Mean ± SD)	Overall improvement rate	Comments/Importance
														
1	Hauser et al., 1999	Clinical trials	37.3 ± 7.9^a^	6	18.2 ± 7.6	3–4 donors per side	Stereotaxy (postcommissural putamen)	Fetal nigral	24 months	−	50.8 ± 17.6/35.4 ± 15.2	32.1 ± 7.1/21.0 ± 7.8	32%	Safely and with little morbidity. long-term clinical benefit is related to the survival and function of transplanted DA neurons.
2	Schumacher et al., 2000	Clinical trials	60.8 ± 6.5^a^	11	14.0 ± 5.9	1.2 × 10^8^ per-side	Stereotaxy (striatal)	Porcine embryonic VM cells	12 months	−	46.6 ± 16.4/39.5 ± 14.0	27.1 ± 6.8/20.4 ± 7.9	19%	Safely and tolerated. No evidence of transmission of porcine pathogens or PERV.
3	Arjona et al., 2003	Clinical trials	53.3^b^	6	11.5	−	Stereotaxy (putamen orcaudate nucleus)	Carotid body	6 months	−	43.3 ± 20.9/30.3 ± 18.5	20.5 ± 9.3/15.8 ± 8.1	32%	safely and tolerable, transplantation of carotid body can improve symptoms and signs in patients with PD.
4	Stover et al., 2005	Clinical trials	52.2^b^	6	10.2	3.25 × 10^5^	Stereotaxy postcommissural putamen (contralateral to affected sideaffected side)	Human retinal pigment epithelial (RPE)	12 months	1 died of depression and suicidal	51.9 ± 8.7/27.2 ± 7.1	24.2 ± 4.5/15.2 ± 5.7	48%	Safely and tolerated, Human RPE cells improved motor symptoms in patients with PD.
5	Mínguez-Castellanos et al., 2007	Clinical trials	52 ± 5^a^	12	11 ± 4	−	Stereotaxy (striatum)	Carotid body	6 months	1 transplant-related lacunar cerebral infarction resulting in sequelae	41.7 ± 15.7/32.3 ± 13.9	20.7 ± 6.9/17.3 ± 5.9	−	CB autotransplantation cannot be considered as a realistic therapeutic option.
6	Venkataramana et al., 2010	Clinical trials	55.4 ± 15.4^a^	7	14.7 ± 7.56	1 × 10^6^ cells/kg body weight	Stereotaxy	Autologous bone-marrow-derived mesenchymal stem cells (BM-MSCs)	12 months	−	65 ± 22.1/22.1 ± 5.8	−	Off: 22.9% On: 38%	Safely. The clinical improvement is only marginal; however, most patients experienced subjective well-being.
7	Gross et al., 2011	RCT	56.4 ± 7.53^a^	32	>5	3.25 × 10^5^ per-side	Stereotaxy (PD postcommissural putamen)	Human retinal pigment epithelial (RPE)	12 months	1 died, possibly related to cell implantation or surgery	48.8 ± 7.74/38.3 ± 10.41	31.4 ± 11.4/27.9 ± 12.0	−	No advantage.
8	Yin et al., 2012	Clinical trials	66 (52–88)^c^	12	>5 (average 6.4)	1 × 10^6^	Stereotaxy (PD postcommissural putamen)	Postmortem (HRPE)	12 months	−	51.8 ± 18.7/27.2 ± 16.4	26.3 ± 10.1/12.7 ± 8.5	25%	Safe and well tolerable, transplantation of hRPE-derived NPCs can improve symptoms and signs in patients with PD.
9	Lige and Zengmin, 2016	Clinical trials	57.33 (42–79)^c^	21	\	Unilaterally 3 × 10^7^	Stereotaxy (striatum)	Neural precursor cells (NPCs)	12–36 months	−	35.5 ± 6.5/33.5 ± 5.3	28.5 ± 8.0/23.6 ± 7.6	−	Transplantation of neural precursor cells may be a valid and safe treatment method for PD.
10	Madrazo et al., 2019	Clinical trials	54.57^b^	7	7.7	8 × 10^6^	Stereotaxy (patients’ dorsal putamina)	Human Neural Progenitor Cells (NPC)	48 months	−	27.6 ± 9.5/19.1 ± 9.0	12.7 ± 4.2/10.3 ± 4.7	−	Safely and no serious adverse events. NPCs are able to stop or slow down the motor deterioration.

*CNS, central nervous system; PERV, porcine endogenous retrovirus; CB, Carotid body; BM-MSCs, bone-marrow-derived mesenchymal stem cells; PD, Parkinson’s Disease; HRPE, human retinal pigment epithelial; NPCs, neural progenitor cells; SD, standard deviation; AEs, adverse events. ^a^Mean ± standard deviation; ^b^Mean; ^c^Median (interquartile range).*

### Meta-Analysis and Main Findings

**FIGURE 3:**
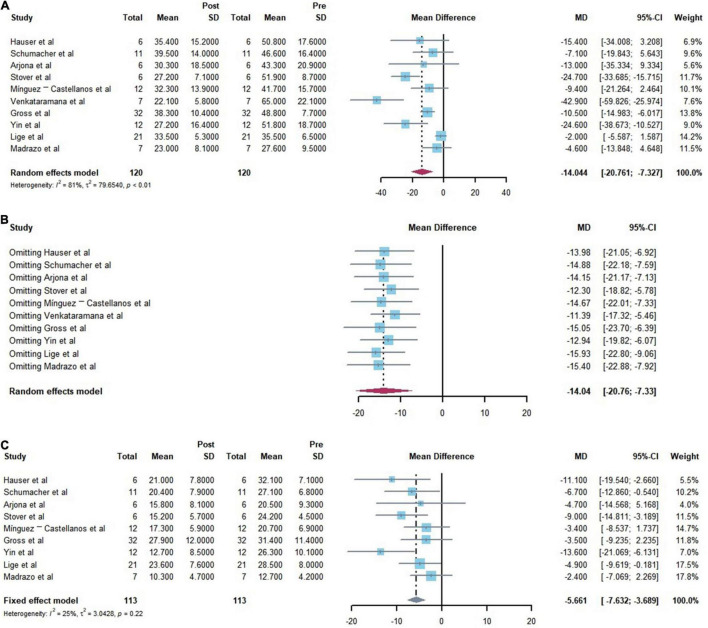
Forest plots of the efficiency of the comparisons before and after cell transplantation. [**(A)** Forest plot for primary outcome analysis on motor function by the Unified Parkinson’s Disease Rating Scale (UPDRS) III. **(B)** Forest plot for Sensitivity analysis of the primary outcomes. **(C)** Forest plot for secondary outcome analysis on daily living ability by the Unified Parkinson’s Disease Rating Scale (UPDRS) II.]

Following a rigorous screening process, 120 patients in 10 studies (9 single-arm clinical trials and 1 RCT studies) were included for analysis. The present meta-analysis aimed at assessing the efficacy and safety of cell transplantation for PD. Regarding efficacy, a significant improvement in all primary outcomes (UPDRSIII) was reported with a score improvement of −14.044 (95% CI: −20.761, −7.327) (*p* < 0.001) for motor. I^2^ index and Cochrane’s Q test were employed to assess the heterogeneity between studies. I^2^ and Q statistics were 81.3% (95% CI: 66.7–89.5%) and 48.19 (*P* < 0.001) (Figure 3A). The studies demonstrated moderate to high heterogeneity. Sensitivity analysis was performed to assess the stability and reliability of our meta-analysis and results revealed that none of the single studies significantly influenced the result (Figure 3B). And a significant improvement in secondary outcome (UPDRSII) was reported with a score improvement of −5.661 (95% CI: −7.632, −3.689) (*p* < 0.001) for ADL. I^2^ and Q statistics were 25% (95% CI: 0–64.6%) and 10.610 (*P* = 0.225), This result illustrates no significant heterogeneity (Figure 3C).

### Subgroup Analysis

The source of heterogeneity was explored by analyzing the impact of the length of disease (<10 years or >10 years) and different follow-up time endpoints (6–12 months or ≥ 18 months) on the effectiveness of cell transplantation. Outcomes based on different mean durations: duration mean < 10 years −8.569 (95% CI: −15.761; −1.376) I^2^ = 80.6%; duration mean > 10 years −18.611 (95% CI: −28.808; −8.414) I^2^ = 67.7%; (subgroup difference test: *P* = 0.115). Outcomes based on different follow-up times: Follow up = 6–12 months −18.061 (95% CI: −26.301; −9.820) I^2^ = 73.7%; Follow up > 18 months −2.912 (95% CI: −6.590; 0.767) I^2^ = 4.6%, (subgroup difference test: *P* < 0.001). Of note, the heterogeneity of the subgroups was reduced, suggesting that these factors are a potential heterogeneity source. More specifically, the best improvement is achieved at nearly 1 year postoperatively (Table 2).

**TABLE 2:** Characteristics of the subgroup analysis of comparisons Before and after cell transplantation according to duration mean and follow-up time.

Outcomes	Index	Subgroup	No. of study	Effect size (95% CI)	I^2^ (%)	*p* values
UPDRS	Disease duration	Duration mean < 10 years	4	MD −8.569 [−15.761; −1.376]	80.6%	0.1147
		Duration mean > 10 years	6	MD −18.611 [−28.808; −8.414]	67.7%	
	Follow-up time	≥ 18 months	3	MD −2.912 [−6.590; −0.767]	4.6%	<0.0010
		6–12 months	7	MD −18.061 [−26.301; −9.820]	73.7%	

*UPDRS, Unified Parkinson’s Disease Rating Scale; MD, mean difference; CI, confidence interval.*

### Evaluation of Publication Bias

The results of Begg’ test for the 10 studies was 0.325 indicating no significant publication bias. Further evaluation of potential publication bias was performed by sensitivity analysis. Similarly, results revealed no obvious bias among studies.

## Discussion

The present meta-analysis of the safety and efficacy of cell transplantation for Parkinson’s disease demonstrated that cell transplantation therapy improves motor function and daily living ability of Parkinson’s patients. The meta-analysis focused on the improvement of UPDRS scores in Parkinson’s patients before and after cell transplantation, whereby the amount of improvement in UPDRSIII and UPDRSII was −14.044 (95% CI: −20.761, −7.327) (*P* < 0.0001), −5.661 (95% CI: −7.632, −3.689) (*P* < 0.0001). Of note, the confidence intervals did not include 0, indicating that the results are statistically significant.

Subgroup analysis demonstrated that compared to follow-up of more than 18 months years, the most improvement of patients occurred at 6–12 months. Among the included studies (Schumacher et al., 2000; Mendez et al., 2005; Mínguez-Castellanos et al., 2007; Kefalopoulou et al., 2014; Madrazo et al., 2019), patients had significantly lower UPDRS (motor) scores with reduced medication. Regarding Efficacy of cell transplantation varies with length of follow-up, the first factor that should be accounted for is the progression of the patient’s Parkinson’s disease, a progressive condition in which motor function declines over time (Huang et al., 2007). The UPDRS assessment revealed the rapid progression of motor dysfunction in Parkinson’s patients in the first 5 years of the disease but slows down in the later stages, with an annual rate of change in UPDRS motor scores of 1.5–3.1% in the off-stage state (Maetzler et al., 2009). These results may be related to cell transplantation survival, dopamine neuron growth, and development.

Moreover, better clinical outcomes after transplantation could be related to patient age and disease duration among the included studies (Freed et al., 2001; Olanow et al., 2003). Although early-stage Parkinson’s patients and mild Parkinson’s patients receive better treatment, attention should also be paid to cases of Parkinson’s subtypes that are unresponsive to drugs. As such, there is a need for awareness on above issues in the future and ensure strict and timely cell transplantation.

Evidence shows that the effect of xenoCell is better than that of allogeneic interspecies cell transplantation, which may be related to multiple factors of cell activity and immune response. Conventional theories suggest that the brain is immunologically privileged, avoiding the concern of graft rejection after allogeneic cell and tissue transplantation. More evidence from primate studies indicates that compared to allografts, autotransplantation of cells promotes better minimization of immune responses in the brain (Morizane et al., 2013). MHC matching is statistically effective at reducing the immune response; however, it does not completely evade the immune response even in the brain (Morizane et al., 2017). On the other hand, the transplantation procedure disrupts the blood-brain barrier, impairs the immune-privileged state of the brain, and may trigger the entry of immune cells (Kim et al., 2013). Therefore, integrating transplantation with immunosuppression in the context of MHC matching may help overcome the potential immune response, in cell transplantation in PD patients and the problem of immune rejection.

Herein, in terms of safety, 7 papers reported that cell transplantation is safe and well-tolerated, whereas 3 papers reported cell transplantation-related complications, including: including depressive suicidal ideation (Stover et al., 2005), cell transplantation-related cerebral infarction (with sequelae) (Mínguez-Castellanos et al., 2007), and cell transplantation procedure-related deaths (Gross et al., 2011). Overall, cellular transplantation therapy is a safe and tolerable treatment but future multicenter studies should be alert on complications related to the procedure. Furthermore, testing the quality of cell sources must be prioritized to assess the viability, state of differentiation, and integrity of genetic material (Stoddard-Bennett and Pera, 2020).

### Transplanted Cell Type

The past four decades have seen the growing adoption of tissue or cell transplantation to manage Parkinson’s disease (Minguez-Castellanos et al., 2007). This approach aims at slowing down neurodegeneration, replacing degenerated dopamine cells, promoting new neuronal connections within the diseased brain, and restoring dopamine secretion. There are various cellular therapies applied to Parkinson’s disease which lack standardized approaches. The first phase focused on human embryonic (Kopyov et al., 1997; Hauser et al., 1999; Schumacher et al., 2000; Freed et al., 2001; Olanow et al., 2003)/pig fetal cell; (Fink et al., 2000; Schumacher et al., 2000; Mulroy et al., 2021)-derived dopamine neurons and retinal pigment epithelial cells designed to release endogenous dopamine and growth factors (Stover et al., 2005; Stover and Watts, 2008).

Fetal neuronal transplantation was the earliest application that achieved neuronal replacement in managing adult CNS disorders. In the initial open clinical trials (human fetus/pig embryo), transplantation of embryo-derived dopaminergic neurons (DA neurons) into PD patients could replace endogenous degenerated DA neurons and improve clinical symptoms of PD (Lindvall, 2013). This approach also demonstrated increased uptake of 18F-DOPA in the transplanted shell nucleus from positron emission tomography (PET) and pathological evidence of survival of dopaminergic neurons and reinnervation of the striatum (Kordower et al., 2008; Mendez et al., 2008). However, ethical, infectious disease, regulatory, and practical issues limit the use of human fetal tissue.

Mesenchymal stem cells are adopted in PD therapy, probably for their neuroprotective potential driven by anti-inflammatory properties and paracrine factors. In the past few years, most researchers purposed to evaluate the positive impact of MSCs in reducing apoptosis and on neuronal cell survival (Andrzejewska et al., 2021). In addition, MSCs reduce the accumulation of polyubiquitinated proteins, contributing to the pathogenesis of transplanted Parkinson’s (Oh et al., 2016). Of note, unlike chronic diseases such as diabetes, patients with Parkinson’s disease have fully functional MSCs, comparable to healthy cells in terms of quantity, quality, plasticity, and immune response (Zhang et al., 2008).

**FIGURE 4:**
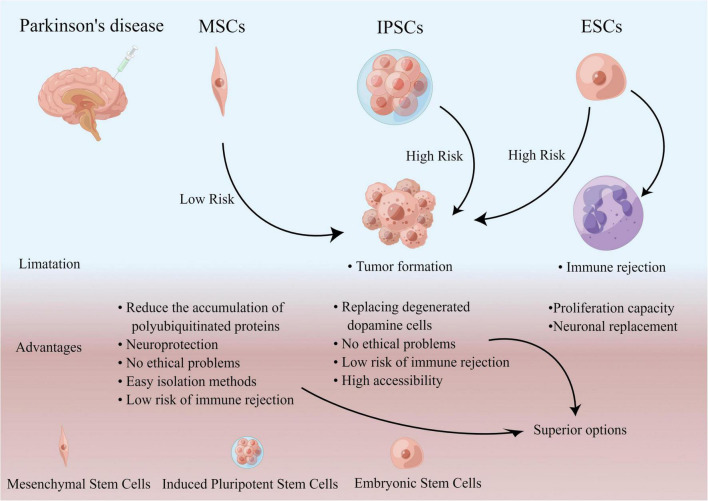
Flow chart of a comparison of the limitation and advantages of cells.

In the future, finished ESC induced iPSC and MSCs may be more superior options compared to previous approaches that used fetal cells (Sonntag et al., 2018; Figure 4).

### Administration Route

The most critical issue in cell therapy protocols is the scheme to determine the optimal dose of cells and the best administration route while considering efficacy and safety. At the same time, the main challenge in cell therapy is the mechanism for the safe and efficient delivery of stem cells to the lesion (De-Munter et al., 2013). Controversy regarding the route of administration also exists. Researchers suggest that intracerebral stereotactic injection of stem cells is precise and efficient but is associated with adverse effects of bleeding or injury-related dysfunction, syncope, and seizures (Olanow et al., 2003). Although nasal administration may reach the central nervous system through the olfactory epithelium and olfactory filaments, potentially avoiding the blood-brain barrier and the capillary network of the lungs and liver, this approach lacks the precision of administration and is accompanied by adverse effects, including nasopharyngeal mucosal damage and allergic reactions. Moreover, the mechanisms of cellular entry into the brain and cellular outcomes are not sufficiently explained and their safety and efficacy have not been validated (Andrzejewska et al., 2021).

Moreover, intravenous administration is relatively safe but systemic injections of cells such as (MSC) are easily captured by cells in the lungs, arriving at the brain in very limited numbers.

For successful intravascular drug delivery, cells must cross the hepatic and pulmonary capillary networks and the blood-brain barrier (BBB) (Andrzejewska et al., 2021), In this view, super-selective arterial catheter insertion techniques for stem cell transplantation may be an ideal delivery method to achieve the release of high concentrations of MSC and selective delivery to arteries in PD-affected brain regions. However, these methods also have disadvantages, including microvascular thrombosis (Brazzini et al., 2010).

Current evidence indicates that cell transplantation is yet to be applied fully in the clinical treatment of Parkinson’s disease. Therefore, more exploration is warranted to fully elucidate the potential mechanisms of cell therapy and provide reliable evidence for the clinical application of cell-based therapies. Accordingly, the present meta-analysis qualitatively and quantitatively evaluated the safety and efficacy of cell transplantation therapy for Parkinson’s disease. The findings suggest that cell transplantation is safe and effective, relative to conventional treatments and hold great application prospect at the forefront of Parkinson’s disease treatment. However, to substantiate the robustness of the findings, studies with larger sample sizes and longer follow-up periods are needed.

### Safety Prospects

Despite promising results regarding efficacy in various clinical trials and proven safety and feasibility, many questions remain unanswered. The cell type, administration route, dose and frequency vary widely between the individual studies. Even among studies for the same disease and using the same cell type, there is large variability between these parameters. all results should be interpreted with caution (Grekhnev et al., 2022; Van-den-Bos et al., 2022). In terms of cell type selection: Transplanted cells should meet criteria regarding safety, efficacy, stability, reproducibility, and scalability before transplantation into patients. Develop a standard for evaluating efficacy after cell transplantation: such as cell survival analyses, neuronal maturation, neurite extension to target cells, formation of synapses with target neurons, and functional integration into the host neural circuitry following cell transplantation (Nishimura and Takahashi, 2016). Monitoring immune reactivity in allogeneic and autologous cell transplantation trials will be key to understanding the role of the immune response to grafted cells and is crucial for future cell therapy development.

## Limitation

A few limitations of this study should be considered. To begin with, some excellent clinical studies could not be included in the clinical analysis due to the lack of raw data. Secondly, limited by the experimental outcome data, the present work only used UPDRS and ADL scores as outcome indicators, which may not completely describe the experimental results. Furthermore, The sample size we used for subgroup analysis was small and there may be false-positive or false-negative results. And the inherent stability of single-arm meta-analysis is lower than that of 2-arm, which is one explanation for the high heterogeneity of this study.

Differences in improvement rates between RCTs and open clinical trials: In studies of cellular therapy for Parkinson’s disease, it is difficult to design a double-blind, identical route of administration, placebo control group when the principle of no harm is to be considered in relation to the potential surgery-inflicted harm to the brain. Differences in improvement rates between RCTs and open clinical trials: According to the principle of no harm, it is difficult to design a double-blind, identical route of administration, placebo control group in studies of cell therapy for Parkinson’s disease that needs to take into account for potential surgery-inflicted harm to the brain. Comparing results of clinical trials to those of randomized controlled trials, the divergence in the efficacy of cell transplantation is wide. Explaining this discrepancy is difficult and can be partly related to differences in the heterogeneity of transplanted tissues, the application of immunosuppressive agents, and follow-up duration. Therefore, extensive research will have to be conducted in the future including elucidating the exact mechanism of action, possible immune rejection, functionality and the survival of the administered cells to draw adequate conclusions. In doing so, controlled or comparative and blinded studies with a large sample size and longer follow-up should be performed using the same cell type, administration route, dose and frequency. Also, for such studies with surgical procedures (including stereotactic surgery), more alert for highly relevant placebo reactions with patients and observers is required (Diederich and Goetz, 2008). An attempt by patients to guess whether they are receiving active study treatment can raise expectations of benefit and create a positive bias toward outcomes. Furthermore, observer-related bias was a crucial factor: baseline scores of patients at enrollment tend to be more severe.

Collectively, the use of a double-blind placebo-controlled design should be considered for future large multicenter studies whenever possible. In case the cell product has to be administered *via* an invasive surgical procedure, ideally a sham-surgical control would be preferred.

## Conclusion

Cellular therapy in Parkinson’s patients is beneficial in improving the motor symptoms of Parkinson’s. However, the effectiveness of this approach warrants further exploration. Meanwhile, in the future, the focus should be to explore the mechanism for screening patients suitable for transplantation, identify cells that are most suitable for Parkinson’s patients, How to implant cells safely and efficiently. To solve this important issue, the development of adequate models reflecting, as accurately as possible, the pathological processes of Parkinson’s disease is required. The iPSCs was a real breakthrough along this path. Our results require additional long-term prospective clinical studies with large samples. With the establishment of best practice guidelines for stem cell therapies, it will be possible to develop more effective and Safety approaches to treating PD.

## Data Availability Statement

The original contributions presented in the study are included in the article/supplementary material, further inquiries can be directed to the corresponding author/s.

## Author Contributions

JW and YT performed the data analyses and wrote the manuscript. XS contributed in literature review and quality assessment of included studies. ZF helped with data extraction and quality assessment of included studies. LJ contributed to the conception of the study, and the manuscript preparation. YH helped to perform the analysis with constructive discussions. All authors contributed to the article and approved the submitted version.

## Conflict of Interest

The authors declare that the research was conducted in the absence of any commercial or financial relationships that could be construed as a potential conflict of interest.

## Publisher’s Note

All claims expressed in this article are solely those of the authors and do not necessarily represent those of their affiliated organizations, or those of the publisher, the editors and the reviewers. Any product that may be evaluated in this article, or claim that may be made by its manufacturer, is not guaranteed or endorsed by the publisher.
